# Supplementary material for: Expected Labor Market Affiliation: A New Method Illustrated by Estimating the Impact of Perceived Stress on Time in Work, Sickness Absence, and Unemployment of 37,605 Danish Employees
Source: Int J Environ Res Public Health. 2021 May 7;18(9):4980. doi: 10.3390/ijerph18094980 (PMC8124718; doi:10.3390/ijerph18094980)
Supplement: Supplementary file 1 [file ijerph-18-04980-s001.zip › ijerph-1182369-supplementary.pdf]

Supplementary material

Table S1A. Expected average number of days (95% confidence interval) spent in the four recurrent labor market affiliation states (ELMA estimates) for women divided by age group.

Women - ELMA 4 year follow-up, estimated in days (95% CI)									
**Age**	**Variable**	**Level**	**Work**	**Sick**	**Unemployment**	**Temp. Out**	**Disability Pension**	**Pension**	**Death**
18-39	Intercept	Reference	1227.4 (1226.2:1228.6)	44.3 (43.9:44.6)	18.0 (17.8:18.3)	134.7 (134.4:135.0)	0.0 (0.0:0.0)	0.0 (0.0:0.0)	0.0 (0.0:0.0)
	Stress	No	. (-)	. (-)	. (-)	. (-)	. (-)	. (-)	. (-)
		Work-related	-50.8 (-52.8:-48.8)	44.6 (43.6:45.6)	12.1 (11.7:12.6)	-11.5 (-12.2:-10.9)	0.0 (0.0:0.0)	0.0 (0.0:0.0)	0.0 (0.0:0.0)
		Work and personal life-related	-61.9 (-64.0:-59.9)	33.8 (32.8:34.7)	5.6 (5.4:5.9)	13.1 (12.6:13.6)	0.0 (0.0:0.0)	0.0 (0.0:0.0)	0.0 (0.0:0.0)
	Education	Low	-36.8 (-38.8:-34.8)	14.7 (13.9:15.4)	11.7 (11.0:12.4)	6.6 (6.0:7.1)	0.0 (0.0:0.0)	0.0 (0.0:0.0)	0.0 (0.0:0.0)
		Middle	. (-)	. (-)	. (-)	. (-)	. (-)	. (-)	. (-)
		High	37.7 (36.7:38.7)	-18.6 (-18.9:-18.3)	-6.8 (-7.0:-6.6)	-11.9 (-12.2:-11.6)	0.0 (0.0:0.0)	0.0 (0.0:0.0)	0.0 (0.0:0.0)
		Not Available	-222.6 (-245.5:-199.6)	180.4 (156.8:204.0)	-10.9 (-12.4:-9.5)	80.9 (70.4:91.4)	0.0 (0.0:0.0)	0.0 (0.0:0.0)	0.0 (0.0:0.0)
	Employment sector	Private	. (-)	. (-)	. (-)	. (-)	. (-)	. (-)	. (-)
		Public	-75.6 (-76.7:-74.5)	29.2 (28.8:29.6)	2.3 (2.1:2.5)	33.4 (33.1:33.6)	0.0 (0.0:0.0)	0.0 (0.0:0.0)	0.0 (0.0:0.0)
		Not Available	2.8 (1.4:4.3)	-0.5 (-1.1:0.1)	13.6 (13.0:14.2)	-9.9 (-10.7:-9.2)	0.0 (0.0:0.0)	0.0 (0.0:0.0)	0.0 (0.0:0.0)
	Work-time arrangement	Full-time	. (-)	. (-)	. (-)	. (-)	. (-)	. (-)	. (-)
		Part-time	-16.2 (-17.2:-15.1)	4.6 (4.2:4.9)	5.3 (5.2:5.4)	16.0 (15.8:16.3)	0.0 (0.0:0.0)	0.0 (0.0:0.0)	0.0 (0.0:0.0)
		Not Available	-166.6 (-168.2:-165.1)	16.6 (16.2:17.0)	44.4 (43.7:45.0)	76.1 (75.5:76.8)	0.0 (0.0:0.0)	0.0 (0.0:0.0)	0.0 (0.0:0.0)
	AH-survey	2012	8.3 (7.2:9.4)	-7.6 (-8.0:-7.3)	8.8 (8.5:9.0)	-5.4 (-5.7:-5.1)	0.0 (0.0:0.0)	0.0 (0.0:0.0)	0.0 (0.0:0.0)
		2014	. (-)	. (-)	. (-)	. (-)	. (-)	. (-)	. (-)
		2012 + 2014	73.8 (72.5:75.2)	-14.4 (-14.9:-14.0)	-2.3 (-2.5:-2.1)	-52.2 (-52.6:-51.8)	0.0 (0.0:0.0)	0.0 (0.0:0.0)	0.0 (0.0:0.0)
40-49	Intercept	Reference	1330.0 (1327.7:1332.3)	61.5 (60.9:62.0)	20.0 (19.6:20.4)	26.1 (25.7:26.4)	3.5 (3.3:3.7)	0.0 (0.0:0.0)	1.0 (0.9:1.2)
	Stress	No	. (-)	. (-)	. (-)	. (-)	. (-)	. (-)	. (-)

		Work-related	-76.7 (-79.8:-73.6)	51.5 (50.2:52.8)	5.7 (5.1:6.3)	13.5 (12.9:14.1)	3.5 (2.9:4.2)	0.0 (0.0:0.0)	-1.5 (-1.7:-1.2)
		Work and personal life-related	-81.3 (-85.2:-77.3)	41.7 (40.6:42.9)	1.9 (1.5:2.4)	1.5 (0.8:2.1)	4.7 (4.2:5.3)	0.0 (0.0:0.0)	6.2 (5.7:6.8)
	Education	Low	-206.7 (-217.4:-196.1)	22.7 (20.5:24.9)	28.7 (27.2:30.2)	0.1 (-0.8:1.0)	10.3 (9.3:11.4)	0.0 (0.0:0.0)	-1.7 (-2.1:-1.3)
		Middle	. (-)	. (-)	. (-)	. (-)	. (-)	. (-)	. (-)
		High	36.1 (34.4:37.8)	-25.2 (-25.6:-24.8)	-9.7 (-9.9:-9.4)	11.9 (11.6:12.2)	-2.5 (-2.7:-2.3)	0.0 (0.0:0.0)	-1.8 (-2.0:-1.6)
		Not Available	-119.9 (-148.7:-91.0)	70.0 (57.5:82.6)	-10.5 (-11.6:-9.5)	-8.7 (-11.0:-6.5)	16.8 (10.2:23.4)	0.0 (0.0:0.0)	15.2 (8.0:22.3)
	Employment sector	Private	. (-)	. (-)	. (-)	. (-)	. (-)	. (-)	. (-)
		Public	-42.7 (-45.3:-40.0)	27.9 (27.3:28.5)	-0.8 (-1.2:-0.5)	-5.4 (-5.8:-5.1)	2.6 (2.3:2.8)	0.0 (0.0:0.0)	4.0 (3.8:4.2)
		Not Available	53.7 (49.2:58.1)	-20.3 (-21.2:-19.3)	-5.9 (-6.5:-5.3)	1.5 (1.0:2.0)	-2.0 (-2.3:-1.6)	0.0 (0.0:0.0)	3.8 (3.5:4.1)
	Work-time arrangement	Full-time	. (-)	. (-)	. (-)	. (-)	. (-)	. (-)	. (-)
		Part-time	-16.7 (-18.5:-15.0)	13.2 (12.8:13.6)	-1.7 (-1.8:-1.5)	1.7 (1.5:1.9)	0.7 (0.5:0.9)	0.0 (0.0:0.0)	-1.3 (-1.4:-1.2)
		Not Available	-413.3 (-430.7:-396.0)	59.9 (55.8:63.9)	56.8 (54.1:59.6)	40.9 (38.5:43.4)	15.9 (14.1:17.7)	0.0 (0.0:0.0)	22.0 (20.2:23.7)
	AH-survey	2012	-7.9 (-10.6:-5.3)	10.3 (9.6:10.9)	-3.5 (-3.8:-3.1)	-5.2 (-5.6:-4.8)	2.6 (2.3:2.8)	0.0 (0.0:0.0)	2.1 (1.9:2.3)
		2014	. (-)	. (-)	. (-)	. (-)	. (-)	. (-)	. (-)
		2012 + 2014	29.9 (27.1:32.7)	-13.7 (-14.2:-13.1)	0.7 (0.3:1.1)	-13.3 (-13.6:-13.0)	-3.8 (-4.0:-3.6)	0.0 (0.0:0.0)	-2.2 (-2.4:-2.1)
50-59	Intercept	Reference	1330.9 (1329.3:1332.5)	33.3 (32.9:33.6)	18.5 (18.3:18.7)	13.1 (13.0:13.3)	0.0 (0.0:0.0)	26.2 (26.0:26.4)	1.3 (1.2:1.4)
	Stress	No	. (-)	. (-)	. (-)	. (-)	. (-)	. (-)	. (-)
		Work-related	-103.1 (-105.7:-100.6)	45.6 (44.8:46.5)	10.6 (10.3:10.8)	-0.4 (-0.7:-0.2)	0.0 (0.0:0.0)	7.8 (7.4:8.2)	2.3 (2.1:2.5)
		Work and personal life-related	-53.0 (-55.8:-50.1)	24.2 (23.6:24.8)	13.1 (12.7:13.4)	-2.9 (-3.1:-2.7)	0.0 (0.0:0.0)	-8.5 (-8.9:-8.2)	-1.9 (-2.1:-1.6)
	Education	Low	-71.3 (-73.7:-69.0)	25.2 (24.6:25.7)	11.1 (10.7:11.4)	-2.3 (-2.5:-2.1)	0.0 (0.0:0.0)	16.2 (15.8:16.6)	2.2 (2.0:2.4)
		Middle	. (-)	. (-)	. (-)	. (-)	. (-)	. (-)	. (-)
		High	19.0 (17.6:20.4)	-3.0 (-3.3:-2.7)	-8.0 (-8.2:-7.8)	0.5 (0.3:0.6)	0.0 (0.0:0.0)	1.0 (0.8:1.2)	-0.4 (-0.5:-0.3)
		Not Available	-2.2 (-28.2:23.9)	-23.7 (-30.2:-17.2)	2.5 (-2.3:7.3)	10.9 (6.4:15.4)	0.0 (0.0:0.0)	-25.4 (-29.3:-21.6)	-2.5 (-4.2:-0.9)
	Employment	Private	. (-)	. (-)	. (-)	. (-)	. (-)	. (-)	. (-)

	sector	Public	-50.8 (-52.6:-49.1)	24.9 (24.6:25.1)	1.3 (1.0:1.5)	8.8 (8.7:8.9)	0.0 (0.0:0.0)	11.8 (11.5:12.0)	0.7 (0.6:0.8)
		Not Available	-5.0 (-7.3:-2.6)	-11.7 (-12.4:-11.0)	2.9 (2.5:3.3)	15.4 (15.1:15.8)	0.0 (0.0:0.0)	5.9 (5.6:6.2)	0.3 (0.1:0.4)
	Work-time arrangement	Full-time	. (-)	. (-)	. (-)	. (-)	. (-)	. (-)	. (-)
		Part-time	-37.4 (-38.5:-36.3)	13.2 (12.9:13.4)	-0.1 (-0.2:0.0)	1.5 (1.4:1.6)	0.0 (0.0:0.0)	26.2 (26.0:26.4)	0.4 (0.3:0.4)
		Not Available	-437.8 (-446.8:-428.7)	50.5 (48.8:52.3)	98.8 (97.1:100.5)	14.5 (13.7:15.3)	0.0 (0.0:0.0)	30.8 (30.1:31.5)	20.4 (19.5:21.4)
	AH-survey	2012	-8.6 (-10.3:-7.0)	5.5 (5.2:5.9)	5.9 (5.7:6.1)	-2.2 (-2.3:-2.0)	0.0 (0.0:0.0)	-0.5 (-0.8:-0.3)	1.6 (1.5:1.7)
		2014	. (-)	. (-)	. (-)	. (-)	. (-)	. (-)	. (-)
		2012 + 2014	26.6 (25.1:28.1)	-0.8 (-1.1:-0.5)	-3.2 (-3.3:-3.0)	-11.2 (-11.3:-11.1)	0.0 (0.0:0.0)	4.8 (4.6:5.1)	-2.0 (-2.1:-1.9)

Table S1B. Expected average number of days (95% confidence interval) spent in the four recurrent labor market affiliation states (ELMA estimates) for men divided by age group.

Men - ELMA 4 year follow-up, estimated in days (95% CI)									
**Age**	**Variable**	**Level**	**Work**	**Sick**	**Unemployment**	**Temp. Out**	**Disability Pension**	**Pension**	**Death**
18-39	Intercept	Reference	1188.8 (1187.4:1190.1)	28.7 (28.6:28.9)	24.2 (23.9:24.5)	90.5 (89.9:91.2)	0.0 (0.0:0.0)	0.0 (0.0:0.0)	-1.5 (-1.8:-1.2)
	Stress	No	. (-)	. (-)	. (-)	. (-)	. (-)	. (-)	. (-)
		Work-related	-37.1 (-40.4:-33.8)	7.3 (7.0:7.6)	10.2 (9.7:10.7)	-16.8 (-18.3:-15.2)	0.0 (0.0:0.0)	0.0 (0.0:0.0)	-3.4 (-4.0:-2.8)
		Work and personal life-related	-46.3 (-49.4:-43.1)	26.7 (25.6:27.8)	24.3 (23.2:25.4)	-21.7 (-23.6:-19.7)	0.0 (0.0:0.0)	0.0 (0.0:0.0)	-0.2 (-0.5:0.1)
	Education	Low	-20.5 (-22.8:-18.2)	10.0 (9.7:10.4)	13.8 (13.2:14.4)	-19.1 (-20.3:-18.0)	0.0 (0.0:0.0)	0.0 (0.0:0.0)	5.4 (4.9:5.9)
		Middle	. (-)	. (-)	. (-)	. (-)	. (-)	. (-)	. (-)
		High	95.3 (93.5:97.0)	-16.1 (-16.3:-15.9)	-14.6 (-14.9:-14.3)	-32.4 (-33.3:-31.6)	0.0 (0.0:0.0)	0.0 (0.0:0.0)	3.2 (3.0:3.5)
		Not Available	-635.6 (-651.1:-620.2)	11.4 (8.3:14.6)	-16.3 (-21.2:-11.4)	558.3 (540.8:575.7)	0.0 (0.0:0.0)	0.0 (0.0:0.0)	-0.4 (-1.3:0.5)
	Employment sector	Private	. (-)	. (-)	. (-)	. (-)	. (-)	. (-)	. (-)
		Public	-125.7 (-127.9:-123.5)	9.5 (9.2:9.8)	9.0 (8.7:9.4)	42.3 (41.1:43.5)	0.0 (0.0:0.0)	0.0 (0.0:0.0)	-0.7 (-0.9:-0.6)
		Not Available	25.1 (23.5:26.8)	-2.7 (-2.9:-2.5)	10.4 (10.0:10.8)	21.7 (21.0:22.5)	0.0 (0.0:0.0)	0.0 (0.0:0.0)	11.9 (11.5:12.3)
	Work-time arrangement	Full-time	. (-)	. (-)	. (-)	. (-)	. (-)	. (-)	. (-)
		Part-time	-130.3 (-133.3:-127.3)	-5.4 (-5.7:-5.1)	13.3 (12.9:13.8)	106.8 (104.7:108.8)	0.0 (0.0:0.0)	0.0 (0.0:0.0)	3.6 (3.1:4.1)
		Not Available	-327.6 (-332.6:-322.6)	5.7 (5.3:6.1)	55.2 (53.7:56.7)	145.0 (142.3:147.6)	0.0 (0.0:0.0)	0.0 (0.0:0.0)	6.6 (5.5:7.6)
	AH-survey	2012	14.2 (12.6:15.7)	-1.0 (-1.2:-0.8)	7.8 (7.5:8.1)	23.5 (22.7:24.3)	0.0 (0.0:0.0)	0.0 (0.0:0.0)	1.0 (0.8:1.3)
		2014	. (-)	. (-)	. (-)	. (-)	. (-)	. (-)	. (-)
		2012 + 2014	119.3 (117.1:121.5)	-5.0 (-5.2:-4.8)	-6.1 (-6.5:-5.8)	-39.9 (-40.9:-38.9)	0.0 (0.0:0.0)	0.0 (0.0:0.0)	-1.3 (-1.5:-1.1)
40-49	Intercept	Reference	1339.4 (1338.4:1340.5)	47.7 (47.5:48.0)	10.9 (10.7:11.1)	22.9 (22.6:23.1)	0.4 (0.3:0.4)	0.0 (0.0:0.0)	0.1 (0.1:0.1)
	Stress	No	. (-)	. (-)	. (-)	. (-)	. (-)	. (-)	. (-)
		Work-related	-37.2 (-39.7:-34.7)	19.0 (18.0:20.0)	1.9 (1.4:2.5)	9.2 (8.2:10.3)	0.2 (0.2:0.2)	0.0 (0.0:0.0)	-0.1 (-0.1:-0.1)
		Work and personal life-related	-117.1 (-122.5:-111.7)	39.8 (38.2:41.4)	18.2 (16.8:19.7)	-0.2 (-0.8:0.3)	0.3 (0.2:0.3)	0.0 (0.0:0.0)	-0.1 (-0.1:-0.1)

	Education	Low	-43.2 (-45.4:-41.0)	15.7 (15.0:16.4)	3.7 (3.2:4.2)	3.3 (2.9:3.7)	-0.2 (-0.2:-0.2)	0.0 (0.0:0.0)	-0.0 (-0.0:-0.0)
		Middle	. (-)	. (-)	. (-)	. (-)	. (-)	. (-)	. (-)
		High	49.6 (48.3:51.0)	-27.4 (-27.8:-26.9)	-4.6 (-4.8:-4.3)	-0.6 (-0.8:-0.4)	-0.3 (-0.3:-0.3)	0.0 (0.0:0.0)	-0.0 (-0.0:-0.0)
		Not Available	-132.7 (-145.0:-120.4)	-26.5 (-29.3:-23.8)	5.9 (4.0:7.8)	135.3 (123.6:147.0)	-0.4 (-0.4:-0.3)	0.0 (0.0:0.0)	0.2 (0.1:0.3)
	Employment sector	Private	. (-)	. (-)	. (-)	. (-)	. (-)	. (-)	. (-)
		Public	-90.1 (-91.9:-88.2)	27.4 (26.7:28.1)	-3.0 (-3.3:-2.7)	2.3 (2.1:2.6)	0.1 (0.1:0.1)	0.0 (0.0:0.0)	0.2 (0.2:0.2)
		Not Available	-12.2 (-13.4:-11.1)	6.1 (5.8:6.4)	-1.4 (-1.7:-1.1)	26.6 (26.2:27.0)	0.3 (0.3:0.3)	0.0 (0.0:0.0)	0.1 (0.1:0.1)
	Work-time arrangement	Full-time	. (-)	. (-)	. (-)	. (-)	. (-)	. (-)	. (-)
		Part-time	-56.5 (-60.6:-52.3)	8.5 (7.4:9.5)	4.4 (4.1:4.7)	8.4 (7.6:9.2)	0.0 (-0.0:0.0)	0.0 (0.0:0.0)	0.3 (0.2:0.3)
		Not Available	-172.9 (-180.1:-165.7)	48.4 (46.1:50.7)	49.4 (46.8:52.0)	10.8 (8.9:12.8)	-0.1 (-0.1:-0.0)	0.0 (0.0:0.0)	-0.1 (-0.2:-0.1)
	AH-survey	2012	-24.5 (-25.8:-23.2)	-0.7 (-1.2:-0.3)	11.5 (11.2:11.8)	10.0 (9.7:10.2)	-0.0 (-0.0:-0.0)	0.0 (0.0:0.0)	0.2 (0.2:0.2)
		2014	. (-)	. (-)	. (-)	. (-)	. (-)	. (-)	. (-)
		2012 + 2014	53.6 (52.1:55.0)	-20.2 (-20.7:-19.8)	-2.7 (-2.9:-2.4)	-13.0 (-13.3:-12.7)	-0.2 (-0.2:-0.2)	0.0 (0.0:0.0)	-0.0 (-0.0:-0.0)
50-59	Intercept	Reference	1316.4 (1315.4:1317.5)	48.9 (48.7:49.1)	8.6 (8.3:8.9)	7.1 (6.9:7.3)	2.6 (2.5:2.6)	4.1 (3.9:4.2)	1.6 (1.5:1.8)
	Stress	No	. (-)	. (-)	. (-)	. (-)	. (-)	. (-)	. (-)
		Work-related	-79.6 (-82.1:-77.1)	22.0 (21.3:22.7)	21.0 (19.9:22.0)	6.8 (6.0:7.6)	3.7 (3.5:3.9)	9.4 (8.8:9.9)	-1.8 (-2.2:-1.3)
		Work and personal life-related	-67.3 (-70.4:-64.2)	45.1 (43.3:46.8)	16.9 (15.5:18.3)	-2.1 (-2.6:-1.5)	3.4 (3.0:3.7)	7.1 (6.5:7.7)	-1.8 (-2.4:-1.1)
	Education	Low	-23.8 (-25.4:-22.2)	3.4 (3.0:3.8)	9.7 (9.2:10.2)	-5.6 (-5.9:-5.3)	0.1 (0.0:0.2)	9.7 (9.4:10.0)	-0.2 (-0.5:0.2)
		Middle	. (-)	. (-)	. (-)	. (-)	. (-)	. (-)	. (-)
		High	21.9 (20.6:23.1)	-22.6 (-22.9:-22.3)	-2.8 (-3.1:-2.5)	11.4 (11.0:11.7)	-1.7 (-1.8:-1.7)	-3.7 (-3.8:-3.6)	-2.2 (-2.5:-1.9)
		Not Available	-35.4 (-54.8:-16.0)	-10.6 (-12.4:-8.8)	28.4 (23.1:33.8)	-3.6 (-4.9:-2.3)	-2.7 (-3.2:-2.1)	-8.2 (-9.2:-7.3)	-0.5 (-1.8:0.8)
	Employment sector	Private	. (-)	. (-)	. (-)	. (-)	. (-)	. (-)	. (-)
		Public	-78.3 (-79.7:-77.0)	18.1 (17.7:18.5)	0.9 (0.5:1.3)	5.0 (4.8:5.2)	2.6 (2.5:2.7)	5.0 (4.8:5.2)	-1.5 (-1.8:-1.2)
		Not Available	17.8 (16.3:19.2)	3.6 (3.4:3.9)	5.7 (5.3:6.2)	16.3 (15.9:16.7)	1.4 (1.3:1.5)	4.2 (4.1:4.4)	-2.3 (-2.6:-1.9)
	Work-time arrangement	Full-time	. (-)	. (-)	. (-)	. (-)	. (-)	. (-)	. (-)
		Part-time	-121.5 (-125.9:-117.1)	12.9 (12.2:13.7)	19.6 (18.6:20.6)	-5.1 (-5.6:-4.5)	3.1 (2.9:3.3)	-1.2 (-1.5:-1.0)	27.8 (25.2:30.4)

		Not Available	-185.1 (-191.6:-178.6)	26.1 (25.0:27.2)	54.9 (52.6:57.3)	-4.0 (-4.5:-3.4)	9.6 (9.2:10.1)	1.0 (0.7:1.2)	-0.3 (-0.4:-0.2)
	AH-survey	2012	-59.0 (-60.3:-57.8)	5.8 (5.5:6.0)	17.5 (17.1:17.9)	5.6 (5.3:5.9)	-0.7 (-0.8:-0.6)	3.9 (3.8:4.0)	1.2 (1.0:1.4)
		2014	. (-)	. (-)	. (-)	. (-)	. (-)	. (-)	. (-)
		2012 + 2014	14.6 (13.4:15.7)	-15.2 (-15.4:-14.9)	-1.9 (-2.2:-1.6)	-7.4 (-7.6:-7.1)	-2.1 (-2.2:-2.0)	8.3 (8.1:8.4)	3.5 (3.1:3.9)
